# Cryopreservation of dermal fibroblasts and keratinocytes in hydroxyethyl starch–based cryoprotectants

**DOI:** 10.1186/s12896-016-0315-4

**Published:** 2016-12-01

**Authors:** Yahaira Naaldijk, Adiv A. Johnson, Annett Friedrich-Stöckigt, Alexandra Stolzing

**Affiliations:** 1 Fraunhofer Institute for Cell Therapy and Immunology, Leipzig, Germany; 2 Interdisciplinary Institute for Bioinformatics, University of Leipzig, Leipzig, Germany; 3 Department of Ophthalmology, Mayo Clinic, Rochester, MN USA; 4 Centre for Biological Engineering, Wolfson School of Material and Manufacturing Engineering, Loughborough University, Loughborough, UK

**Keywords:** Fibroblasts, Keratinocytes, Cryoprotectants, Hydroxyethyl starch, Dimethyl sulfoxide, Cryopreservation

## Abstract

**Background:**

Preservation of human skin fibroblasts and keratinocytes is essential for the creation of skin tissue banks. For successful cryopreservation of cells, selection of an appropriate cryoprotectant agent (CPA) is imperative. The aim of this study was to identify CPAs that minimize toxic effects and allow for the preservation of human fibroblasts and keratinocytes in suspension and in monolayers.

**Results:**

We cryopreserved human fibroblasts and keratinocytes with different CPAs and compared them to fresh, unfrozen cells. Cells were frozen in the presence and absence of hydroxyethyl starch (HES) or dimethyl sulfoxide (DMSO), the latter of which is a commonly used CPA known to exert toxic effects on cells. Cell numbers were counted immediately post-thaw as well as three days after thawing. Cellular structures were analyzed and counted by labeling nuclei, mitochondria, and actin filaments. We found that successful cryopreservation of suspended or adherent keratinocytes can be accomplished with a 10% HES or a 5% HES, 5% DMSO solution. Cell viability of fibroblasts cryopreserved in suspension was maintained with 10% HES or 5% HES, 5% DMSO solutions. Adherent, cryopreserved fibroblasts were successfully maintained with a 5% HES, 5% DMSO solution.

**Conclusion:**

We conclude that skin tissue cells can be effectively cryopreserved by substituting all or a portion of DMSO with HES. Given that DMSO is the most commonly used CPA and is believed to be more toxic than HES, these findings are of clinical significance for tissue-based replacement therapies. Therapies that require the use of keratinocyte and fibroblast cells, such as those aimed at treating skin wounds or skin burns, may be optimized by substituting a portion or all of DMSO with HES during cryopreservation protocols.

## Background

Tissue engineering employs cells, biomaterials, and engineering to repair damaged tissue, replace missing tissue, and/or enhance the function of existing tissue [1]. Tissue engineering shows particular promise for victims of skin wounds and skin burns. One clinical approach for these patients is tissue-based replacement therapy, which can utilize cryopreserved dermal fibroblast and keratinocyte cells to repair human skin [2]. Cryopreservation, by providing on demand, pretested cells produced in large, standardized batches, has many clinical advantages. This therapeutic approach hinges on a cryopreservation protocol which optimally preserves the health and function of skin fibroblasts and keratinocytes.

There is an unmet need for the optimization and development of more efficient protocols that preserve cellular integrity. In order to enhance cell survival during and after cryopreservation, cryoprotectant agents (CPA) are used. Many standard cryopreservation methods utilize fetal calf serum (FCS) and/or dimethyl sulfoxide (DMSO). While effective, FCS is limited in that it is an animal product and therefore has a probability of contamination (e.g., with bacteria, viruses, or prions) [3]. DMSO is the most standard CPA used but is disadvantaged in that it exhibits toxicity to cells in vitro as well as in patients following clinical application. At low concentrations, DMSO thins cell membranes and increases their fluidity [4]. At higher concentrations, DMSO induces the formation of transient water pores and can prompt disintegration of the lipid bilayer [4]. Clinically, the infusion of peripheral blood progenitors cryopreserved using DMSO was reported to cause minor to moderate toxicity in patients and the grade of toxicity was correlated with the amount of DMSO present in the transplanted graft [5]. Symptoms of toxicity included vomiting, nausea, hypotension, and hypertension with tachycardia [5]. While the most common toxic side effects associated with DMSO following transplantation affect the respiratory and cardiovascular systems [6], neurotoxicity following infusion of DMSO-cryopreserved peripheral blood stem cells has also been reported [7]. DMSO-associated toxicity in adult and pediatric recipients of transplanted, cryopreserved cells has been reported by numerous laboratories [8–13].

Hydroxyethyl starch (HES) is another CPA that is used as a plasma substitute in the clinical setting for the treatment of blood loss caused by hemorrhage, burns, and other tissues injuries [14, 15]. When used at reasonable concentrations, HES is free of side effects and appears to be less toxic than DMSO [16–19]. There are several publications that illustrate the use of HES in cell cryopreservation. HES has been previously used to cryopreserve keratinocytes [20, 21], islets [22], red blood cells [23, 24], peripheral blood stem cells [25, 26], and other cell types [27, 28]. The addition of HES to the CPA solution has been reported to increase the recovery and viability rate after freezing [21]. In our recent work, we successfully cryopreserved rat mesenchymal stem cells using a 5% DMSO, 5% HES solution [29]. Like any other CPA, the efficacy of HES in cryopreservation might depend on the freezing protocols employed, the techniques and materials used, and the cell type being preserved.

In the present study we compare different CPA solutions where DMSO was either reduced or substituted with HES. Following cryopreservation and thawing in different CPAs, we analyzed viability and proliferation of the keratinocyte cell line HaCaT, the fibroblast cell line BJ, and primary foreskin fibroblasts. To our knowledge, no group has yet tested the ability of HES to cryopreserve skin fibroblasts. We find that successful cryopreservation of both keratinocytes and fibroblasts can be achieved by using HES as a CPA.

## Methods

### Cell sources

Primary human fibroblast cells were isolated from foreskin tissue obtained from the Children’s Hospital in Leipzig, Germany. Ethical approval was obtained from the University of Leipzig Ethics Commission. Written consent was obtained from all patients regarding sample collection. The human keratinocyte cell line HaCaT was purchased from Cell Lines Service while the human fibroblast cell line BJ was obtained from ATCC. Consent was obtained from the patients’ parents/guardians and the consent was informed.

## Cell culture

HaCaT, BJ, and primary human fibroblast cells were cultured in DMEM 1X containing high glucose (Gibco) supplemented with 1% penicillin/streptomycin (Gibco) and 10% FCS (Perbio). Cells were trypsinized at 90% confluency with trypsin/EDTA 1X (Gibco) and centrifuged for 5 min at 1000 rpm prior to subsequent cryopreservation or subculture.

### Cryopreservation procedure

As we have done previously [29, 30], different CPA combinations containing DMSO (Sigma), serum (Hyclone), HES (Serumwerke), and/or DMEM (Gibco) were used to generate desired cryosolutions (Table 1). A controlled-rate freezing system (Thermo Scientific Model 7452 Series) was used to cryopreserve the cells. Detailed cryopreservation protocols and procedures for cryopreserving HaCaT, BJ, and primary human fibroblast cells have been described in our previous work [30]. Fresh, non-cryopreserved cells were used as a control for each experiment. Fresh cells for monolayer cryopreservation were seeded at 50,000 cells per well in a 24-well plate and subjected to 3 additional days in culture. For cells cryopreserved in suspension, fresh cells were seeded at 10,000 cells per well in a 24-well plate and cultivated for 3 days.

**Table 1:** Cryoprotectant solutions

Cryoprotectants	
10% DMSO + 90% FCS	
5% DMSO + 95% FCS	
5% DMSO + 5% HES + 90% FCS	
10% HES + 90% FCS	
10% HES + 90% DMEM	
10% DMSO + 90% DMEM	

### Post-thaw cell count and MTT Assay

The protocol employed for post-thaw cell counts as well as use of the MTT assay has been described in our previous work [30]. Media was not changed and cells were not re-plated during the three-day post-thaw period. Prior to measuring cell viability, dead cells were removed by replacing the medium with fresh medium containing MTT.

### Cellular staining

Mitochondria were stained using MitoTracker® CMX-Ros (Invitrogen). A stock of 1 mM MitoTracker was diluted to 200 nM in standard culture media. Media was removed from the cells and replaced with media containing 200 nM MitoTracker. Cells were incubated for 30 min at 37 °C, washed once with PBS 1X, and fixed with 4% paraformaldehyde (PFA) for 15 min at 4 °C. Cells were washed three times with PBS 1X and subsequently stained for actin and nuclei using phalloidin (1:1000; Invitrogen) and DAPI (1:10000; Sigma), respectively. Cells were stained in PBS 1X containing 1% Triton-X for 20 min at room temperature. Cells were washed once with PBS 1X and observed under a fluorescent microscope. Pictures were taken using a Leica System.

### Flow cytometry

We utilized flow cytometry to quantify the cellular staining of mitochondria and actin. Following mitochondrial staining using MitoTracker, cells were trypsinized with trypsin/EDTA 1X and centrifuged at 1000 rpm for 5 min. Stained cells were subsequently fixed with 4%PFA for 15 min at 4 °C and then washed twice with PBS 1X. Then, cells were stained with phalloidin and washed twice with PBS 1X. Samples were then run on a BD Accuri C6 Flow Cytometer (BD Biosciences) and 10,000 cells were counted. Samples were analyzed using FCS Express Software.

### Statistics

For each experiment involving BJ and HaCaT cells, the presented data represents the mean of 3–5 independent experiments. For each experiment involving primary fibroblasts, the presented data represents the mean of 3–5 independent donors. Error bars represent the standard error of the mean (SEM). SigmaPlot software was used to perform one way analysis of variance (ANOVA) followed by Tukey’s test. Statistical significance was defined as *p* < 0.05.

## Results

### Post-thaw cell counts of human keratinocytes and fibroblasts following cryopreservation in different CPA solutions

**Fig. 1:**
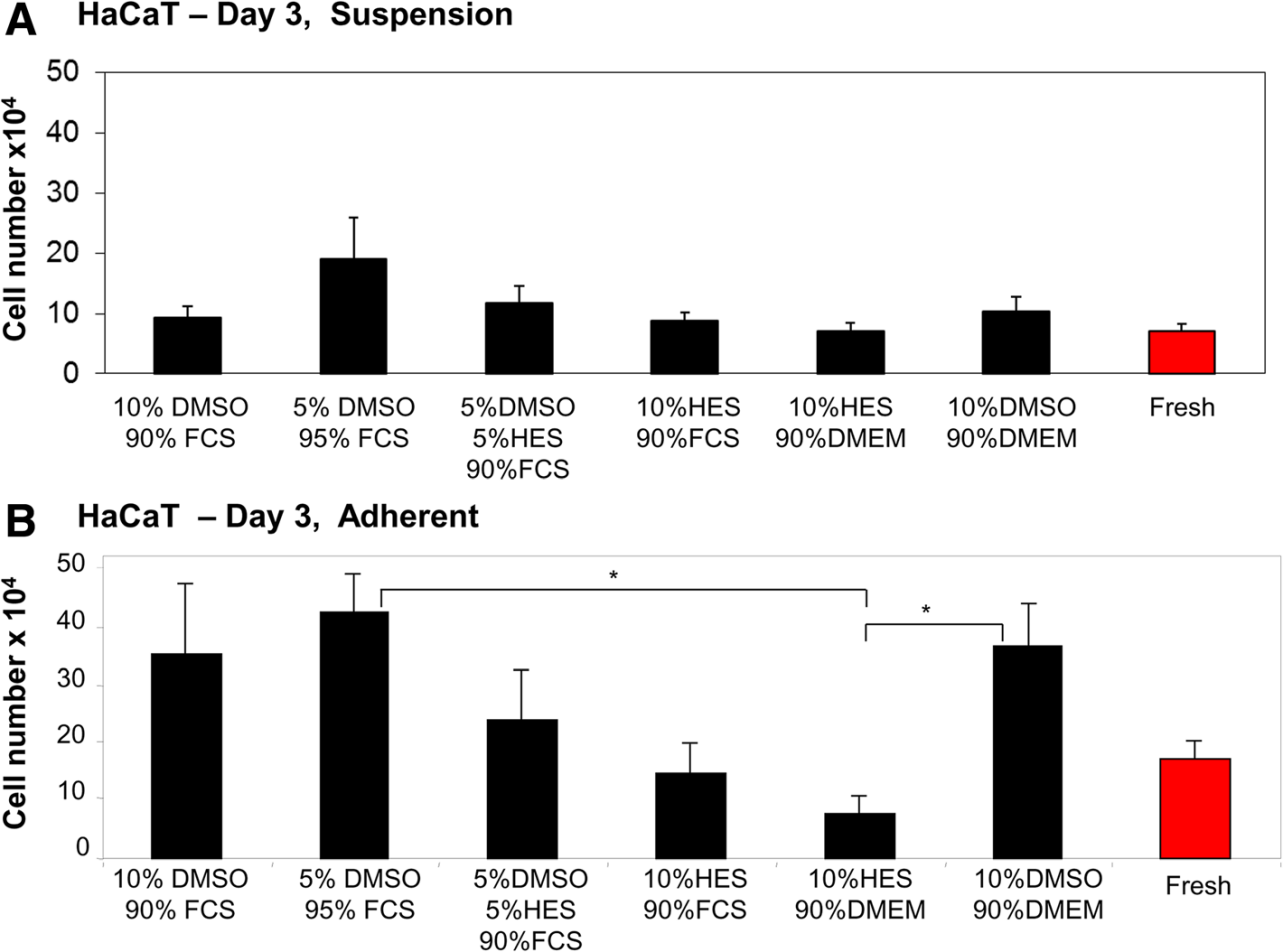
Cryopreservation of HaCaT keratinocyte cells using different cryoprotectants. HaCaT cells were cryopreserved in different cryoprotective solutions. The total number of cryopreserved HaCaT cells in suspension **a** or in adherent monolayers **b** was counted 3 days after thawing. As a comparison, fresh HaCaT cells were counted. * indicates *p* < 0.05

As described in the Methods, HaCaT cells in either suspension (Fig. 1a) or in monolayers (Fig. 1b) were cryopreserved using different combinations of the CPAs DMSO, HES, and FCS (Table 1). Cell counts were made three days post-thaw and compared to the number of cells in fresh, unfrozen cells (Fig. 1).

For HaCaT cells cryopreserved in suspension, the cell number was statistically comparable between fresh cells and cells thawed in various CPA combinations (Fig. 1a). For adherent HaCaT cells, the cell number was significantly lower in cells cryopreserved in a 10% HES, 90% DMEM solution than in cells cryopreserved in either a 5% DMSO, 95% FCS solution or a 10% DMSO, 90% DMEM solution (Fig. 1b).

**Fig. 2:**
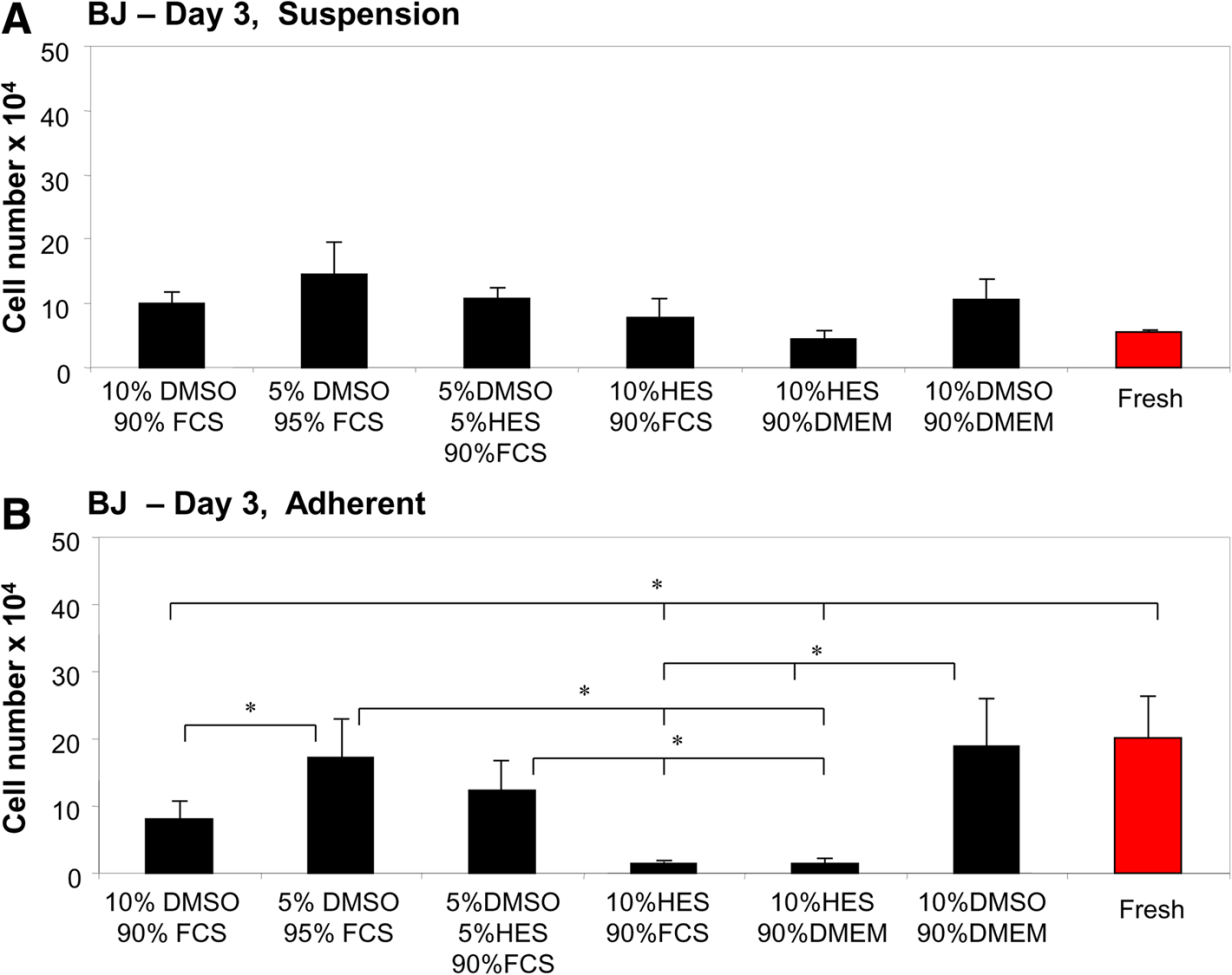
Cryopreservation of BJ fibroblast cells using different cryoprotectants. BJ cells were cryopreserved in different cryoprotective solutions. The total number of cryopreserved BJ cells in suspension **a** or in adherent monolayers **b** was counted 3 days after thawing. As a comparison, fresh BJ cells were counted. * indicates *p* < 0.05

For BJ cells cryopreserved in suspension, cell counts were statistically comparable between fresh BJ cells and BJ cells thawed in various CPA solutions (Fig. 2a). In contrast, adherent BJ cells cryopreserved in a 10% DMSO, 90% FCS solution, a 10% HES, 90% FCS solution, or a 10% HES, 90% DMEM solution showed significantly reduced cell counts compared to fresh cells (Fig. 2b). The 5% DMSO, 95% FCS solution, the 5% DMSO, 5% HES, 90% FCS solution, and the 10% DMSO, 90% DMEM solution displayed significantly higher cell counts than the 10% HES, 90% FCS and 10% HES, 90% DMEM solutions (Fig. 2b).

**Fig. 3:**
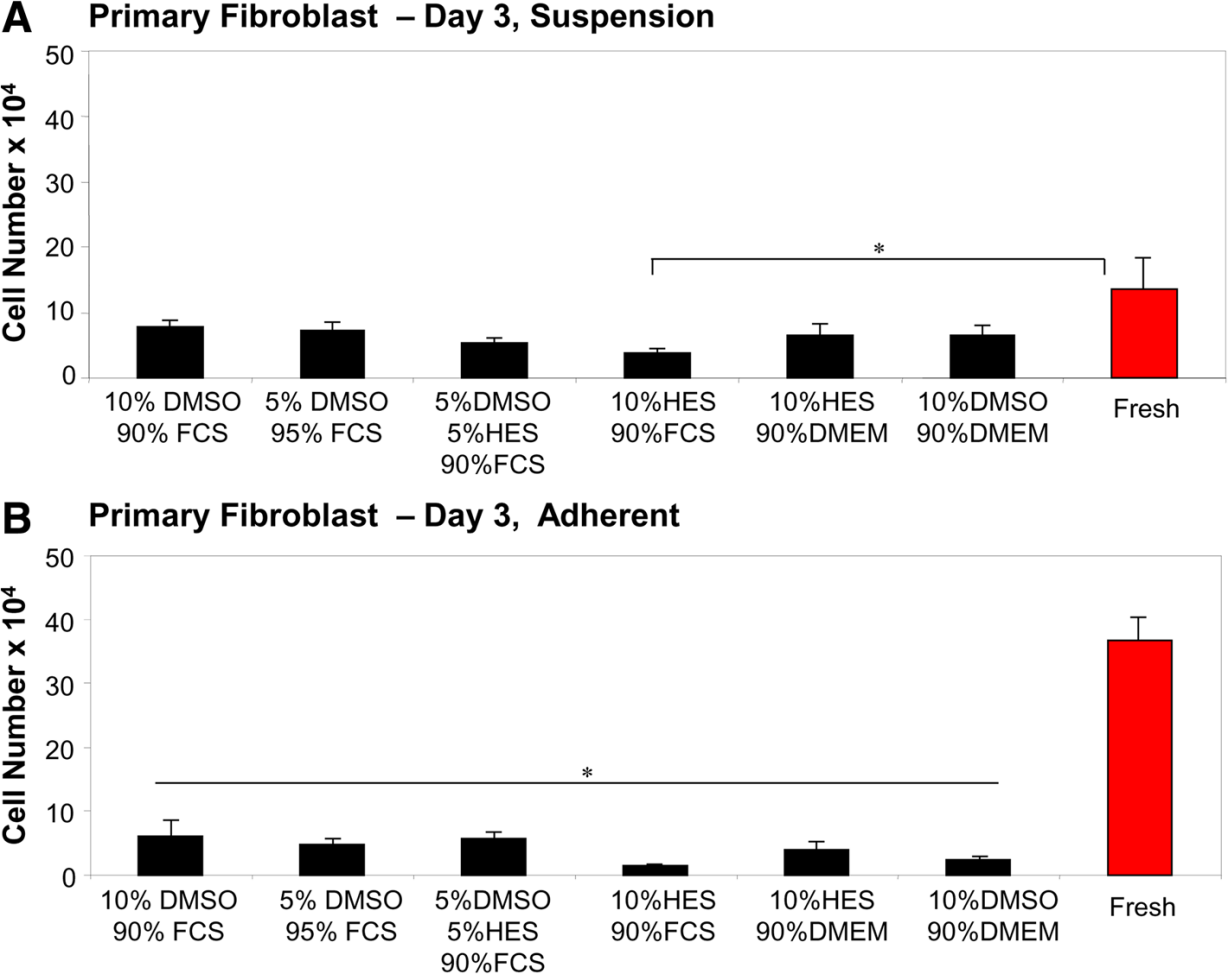
Cryopreservation of primary fibroblast cells using different cryoprotectants. Primary fibroblast cells were cryopreserved in different cryoprotective solutions. The total number of cryopreserved primary fibroblast cells in suspension **a** or in adherent monolayers **b** was counted 3 days after thawing. As a comparison, fresh primary fibroblast cells were counted. * indicates *p* < 0.05

The cell number for primary fibroblasts frozen in suspension was significantly reduced for the 10% HES, 90% FCS solution compared to fresh cells (Fig. 3a). All other solutions exhibited comparable cell counts to fresh cells (Fig. 3a). Regardless of the CPA solution used, all cryopreserved, adherent primary fibroblasts showed a significantly reduced cell number compared to unfrozen cells (Fig. 3b). Table 2 summarizes the total cell numbers for HaCaT, BJ, and primary fibroblast cells 3 days post-thaw.

**Table 2:** Cell viability of each cryopreservation solution for both keratinocytes and fibroblasts on Day 3. Numbers are reported as mean cell number

Cell Type	10% DMSO + 90% FCS	5% DMSO + 95% FCS	5% DMSO + 5% HES + 90% FCS	10% HES + 90% FCS	10% HES + 90% DMEM	10% DMSO + 90% DMEM	Fresh
HaCaT Suspension	93328	217208	117635	88089	71273	103816	70383
HaCaT Adherent	360294	436032	244156	151272	80313	376274	177079
BJ Suspension	100002	130181	107568	76172	40912	131993	54945
BJ Adherent	80448	168692	123244	14801	15238	189289	202214
Primary Fibroblast Suspension	77990	73114	53455	37670	65406	63977	136237
Primary Fibroblast Adherent	60997	58264	57475	14271	39625	23652	36170

**Fig. 4:**
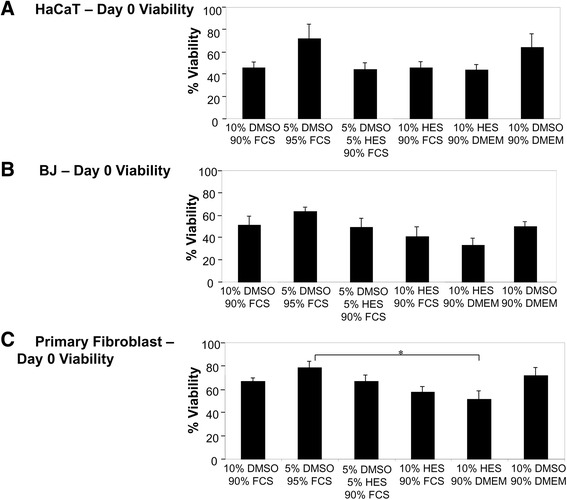
Viability of cryopreserved HaCaT, BJ, and primary fibroblast cells immediately post-thaw on Day 0. HaCaT, BJ, and primary fibroblast cells were cryopreserved in different cryoprotective solutions. Immediately post-thaw on Day 0, percent cell viability was calculated for each cell type and condition. * indicates *p* < 0.05

For both HaCaT (Fig. 4a) and BJ (Fig. 4b) cells, no significant differences in percentage cell viability were observed immediately post-thaw on Day 0. The only significant difference observed was in primary fibroblast cells, where cells frozen in a 10% HES, 90% DMEM solution showed a significantly reduced cell viability (*p* = 0.018) compared to cells frozen in a 5% DMSO, 95% FCS solution (Fig. 4c).

### Cellular staining of human keratinocytes and fibroblasts following cryopreservation in different CPA solutions

Cellular integrity of HaCaT, BJ, and primary fibroblasts cells preserved as monolayers was further assessed via cellular staining of nuclei, actin, and mitochondria following cryofreezing using different CPA solutions.

**Fig. 5:**
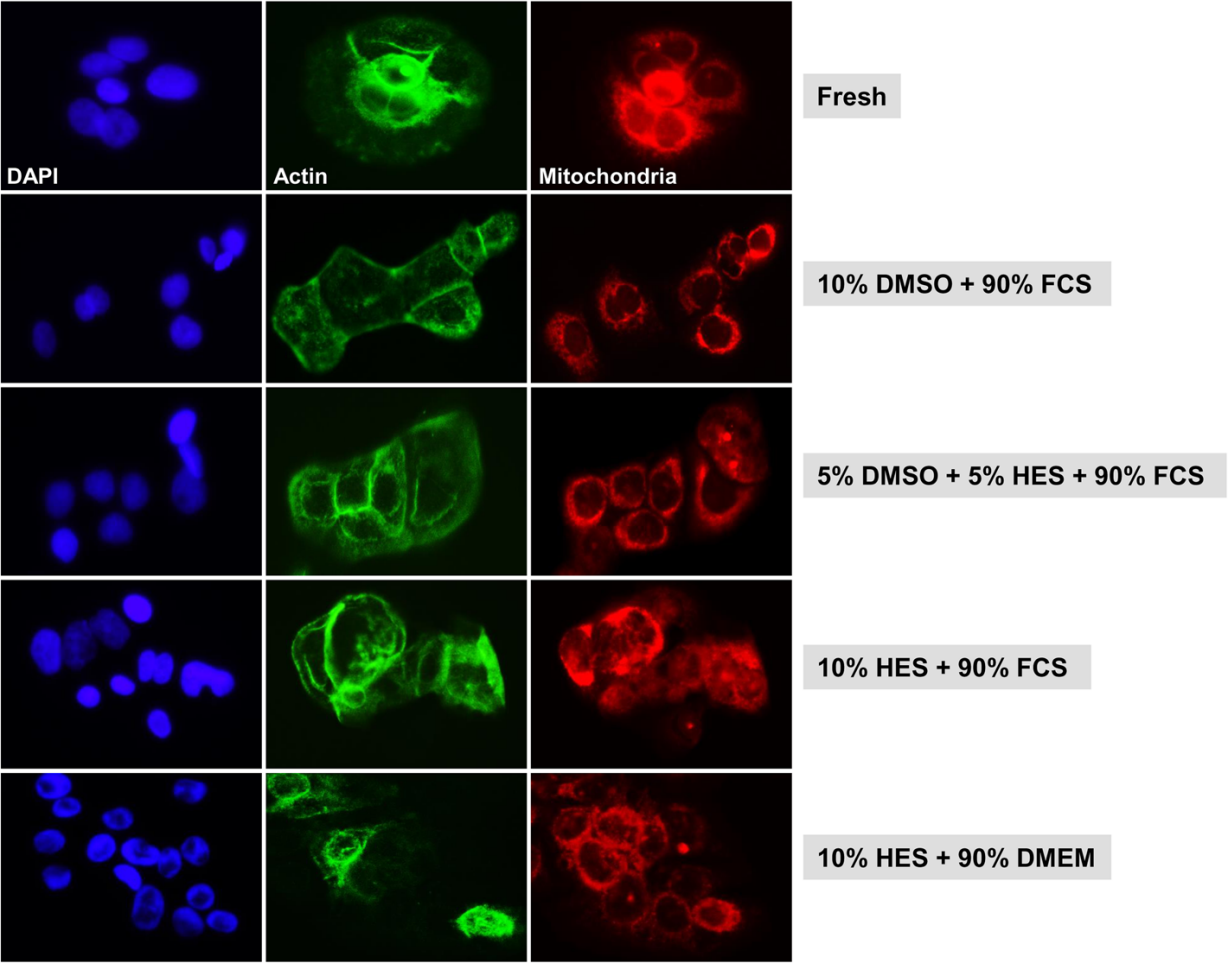
Cellular staining of HaCaT keratinocyte cells preserved as a monolayer. Representative immunofluorescence images are shown for cryopreserved HaCaT cells preserved as monolayers in different cryoprotective solutions. Three days after thawing, cells were stained for nuclei, actin, and mitochondria. For comparison, nuclei, actin, and mitochondria were stained in fresh HaCaT cells

We first compared the immunofluorescence staining of HaCaT cells cryopreserved as monolayers to fresh HaCaT cells (Fig. 5). We found that mitochondrial and actin fluorescence were normal or near-normal in fresh cells as well as in thawed cells cryopreserved with a 10% DMSO and 90% FCS solution, a 5% DMSO, 5% HES, and 90% FCS solution, or a 10% HES and 90% FCS solution. The actin and mitochondrial staining was anomalous or reduced in many keratinocytes cryopreserved with a 10% HES and 90% DMEM solution (Fig. 5).

**Fig. 6:**
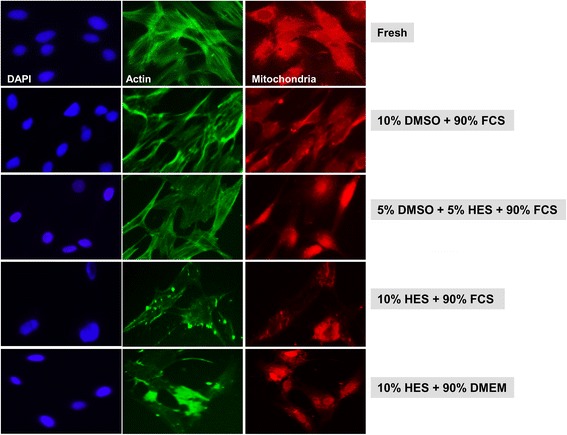
Cellular staining of BJ fibroblast cells preserved as a monolayer. Representative immunofluorescence images are shown for cryopreserved BJ cells preserved as monolayers in different cryoprotective solutions. Three days after thawing, cells were stained for nuclei, actin, and mitochondria. For comparison, nuclei, actin, and mitochondria were stained in fresh BJ cells

Fresh, BJ cells or BJ cells cryopreserved in a 10% DMSO and 90% FCS solution or a 5% DMSO, 5% HES, and 90% FCS solution showed normal actin staining (Fig. 6). BJ cells cryopreserved in either solution exhibited slightly odd mitochondrial staining compared to fresh cells. Both actin and mitochondria appeared abnormal or absent in BJ cells cryopreserved in 10% HES and 90% FCS or 10% FCS and 90% DMEM solutions (Fig. 6).

**Fig. 7:**
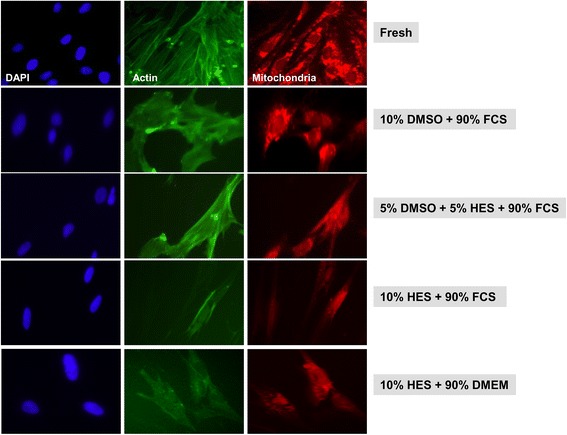
Cellular staining of primary fibroblast cells preserved as a monolayer. Representative immunofluorescence images are shown for cryopreserved primary fibroblast cells preserved as monolayers in different cryoprotective solutions. Three days after thawing, cells were stained for nuclei, actin, and mitochondria. For comparison, nuclei, actin, and mitochondria were stained in fresh primary fibroblast cells

Fresh, primary fibroblasts grown in monolayers showed robust staining for actin and mitochondria (Fig. 7). Although the overall cell numbers were significantly reduced (Fig. 3), actin and mitochondria appeared normal or well-nigh normal in adherent, primary fibroblasts cryopreserved with a 10% DMSO, 90% FCS solution or a 5% DMSO, 5% HES, 90% FCS solution (Fig. 7). Both actin and mitochondrial staining were absent or appeared dysfunctional in primary fibroblasts cryopreserved with solutions containing 10% HES (Fig. 7).

**Fig. 8:**
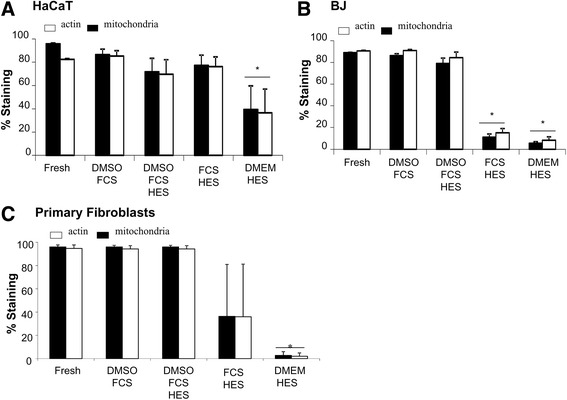
Quantification of actin and mitochondria labeling by flow cytometry. HaCaT, BJ, and primary fibroblast cells were cryopreserved in different cryoprotective solutions. Three days after thawing, cells were stained for nuclei, actin, and mitochondria. Flow cytometry analysis was performed and the percentage of HaCaT **a**, BJ **b**, and primary fibroblast **c** cells showing staining for actin and mitochondria was counted. * indicates *p* < 0.05 compared to fresh cells

Figure 8 summarizes the immunofluorescence data for HaCaT, BJ, and primary fibroblast cells. The percentage of cells with coherent staining for actin and mitochondria was significantly reduced in HaCaT cells cryopreserved in a 10% HES and 90% DMEM solution (Fig. 8a). Significantly fewer BJ cells cryopreserved in a 10% HES, 90% FCS or a 10% HES, 90% DMEM showed staining for actin and mitochondria (Fig. 8b). For primary fibroblast cells, a statistically significant reduction in percent staining for actin and mitochondria was observed for cells cryopreserved in a 10% HES, 90% DMEM solution. A reduction was also observed in primary fibroblast cells cryopreserved in a 10% HES, 90% FCS solution, but this reduction was not statistically significant (Fig. 8c).

## Discussion

Clinical approaches in regenerative medicine frequently involve the transplantation of cells, tissue constructs, and organs [31]. While many studies have worked to optimize the culturing of live cells prior to transplantation [32–34], a major obstacle in this field is the distribution and timing of finite products for clinical use [35]. As allogenic products are becoming more frequently used, it is paramount to optimize the long-term storage of cells and tissues to help minimize clinical costs and make the distribution easier.

We and others have demonstrated that the successful cryopreservation of different human cell types can be accomplished using HES alone or in combination with DMSO [16]. It was previously shown by Pasch et al. that HES can be used to optimally cryopreserve human keratinocytes in suspension and in monolayers [20, 21]. Our data for HaCaT cells corroborate these findings, showing that both cell numbers (Fig. 1) and cellular structures (Fig. 8) are comparable to fresh cells following cryopreservation with HES. Table 3 summarizes the optimal cryopreservation solution containing HES identified for each cell type. For keratinocytes cryopreserved in either suspension or in monolayers, both the 5% DMSO, 5% HES, 90% FCS and the 10% HES, 90% FCS solutions maintained cell viability (Table 3).

**Table 3:** The optimal cryopreservation solutions containing HES identified for keratinocytes and fibroblasts

Cell Type	Suspension	Monolayer
HaCaT	5% DMSO + 5% HES + 90% FCS or 10% HES + 90% FCS	5% DMSO + 5% HES + 90% FCS or 10% HES + 90% FCS
BJ	5% DMSO + 5% HES + 90% FCS or 10% HES + 90% FCS	5% DMSO + 5% HES + 90% FCS
Primary fibroblasts	5% DMSO + 5% HES + 90% FCS	N/A

We previously identified the ideal freezing protocols to use for the cryopreservation of human keratinocytes and fibroblasts [30]. To our knowledge, however, no group has compared cryopreservation efficacy in fibroblasts preserved with HES versus other CPAs. As assessed by measuring cell numbers (Fig. 2) and analyzing nuclei, actin, and mitochondria (Fig. 8), cryopreservation of BJ fibroblasts in suspension was successfully achieved using a 5% DMSO, 5% HES, 90% FCS or a 10% HES, 90% FCS solution (Table 3). BJ cells in monolayers were cryopreserved well in a 5% DMSO, 5% HES, 90% FCS solution (Table 3). In contrast, primary fibroblasts in monolayers showed decreased cell viability when cryopreserved in any solution containing HES (Figs. 3 and 8). Primary fibroblasts in suspension could be successfully cryopreserved in a 5% DMSO, 5% HES, 90% FCS solution (Table 3).

Significant differences in cell viability were observed between HaCaT, BJ, and primary fibroblast cells (Figs. 1, 2, and 3). During cryopreservation, cells and tissues must traverse a lethality zone of temperature (−15 to −60 °C) when being frozen down to very low temperatures and then once again when being thawed [36]. While passing through this zone, cells and tissues must endure the damaging effects of vitrification, cold shock, osmotic injury, and intracellular ice formation [36]. Our data indicate that different cell types are uniquely capable of resisting and repairing damage that occurs during cryopreservation and subsequent thawing. Of particular interest is that the viability of BJ cell line fibroblasts (Fig. 2) was notably higher than the viability of primary fibroblasts (Fig. 3). One possibility is that, since BJ cells are commercially available and routinely undergo freeze-thaw cycles, this cell line has undergone a selection for cells that can robustly withstand cryopreservation. In contrast, primary fibroblasts derived from donors have had no such opportunity to undergo a selection for cells especially resistant to freezing-induced damage. HaCaT cells seemed to cryopreserve more efficiently than fibroblast cells. Since keratinocytes are smaller in size than fibroblasts [37, 38] and cells tend to shrink due to osmosis during cryopreservation [39], a potential explanation for this is difference is that fibroblasts undergo more drastic cell shrinkage during freezing and subsequent thawing. This proposed increase in cell shrinkage stress may result in decreased viability.

As we have previously found with mesenchymal stem cells [29], predominantly negligible differences in cell viability were found immediately post-thawing at Day 0 for HaCaT, BJ, and primary fibroblast cells (Fig. 4). This is most likely explained by the fact that cryopreservation-induced apoptotic cell death has been estimated to take between 6 and 24 h [40] and can still be observed 24-h post-thaw [41]. We chose not to include additional time points beyond Day 3 as, in our previous work with mesenchymal stem cells [29], we found minimal differences in cell viability between Day 3 and Day 14-post-thaw. Interestingly, the cell number for some cryopreservation conditions in HaCaT cells was higher than the control, fresh cells (Fig. 1). It is possible that, for certain cell types, specific CPA solutions promote viability and growth over fresh cells. The general variations in cell number are due to each cell type having a unique proliferation and survival rate in adherent vs. suspension conditions.

It should be noted that, while HES is thought to be clinically safe [16], toxic effects of HES have been noted in the literature. Singbartl et al. reported that cryopreservation of erythrocytes using HES in lieu of glycerol led to the development of transient rheological alterations, including an altered membrane skeleton [42]. A systematic review assessing the use of HES for fluid management found that HES may increase the risk of acute renal failure in patients with sepsis [43]. This risk appeared to increase with higher doses of HES [43]. While HES has been reported in association with adverse effects, a much higher of concentration of HES is required to induce toxicity than DMSO [16].

A randomized phase III clinical trial found that autologous blood stem cell transplantation could be effectively accomplished using cells cryopreserved in a 5% DMSO, 6% HES solution or a 10% DMSO solution. While two patients infused with cells cryopreserved with DMSO displayed serious neurological toxicity, none of the patients who received DMSO/HES cryopreserved cells showed any serious toxicities [44]. Given concerns of DMSO toxicity and the low cost of HES, the authors of that trial suggested replacing a portion of DMSO with HES when cryopreserving cells [44]. A literature review summarizing the known data regarding HES corroborates this trial data, suggesting that HES is less toxic than DMSO and that, at low concentrations, HES is clinically safe [16]. Although the data seem to suggest that DMSO is more toxic than HES, further studies should be performed to specifically compare the clinical safety of HES vs DMSO in patients transplanted or infused with cryopreserved cells.

While we feel that the data contained herein are novel and significant, there are limitations of this work. Ideally, the concentration of FCS would be decreased or FCS would be omitted entirely since it is a derived animal product and therefore susceptible to contamination. Although we did include two different CPA combinations that omitted FCS entirely (10% HES, 90% DMEM and 10% DMSO, 90% DMEM), the remaining CPA combinations only featured two different concentrations of FCS – 90% and 95%. Future studies are warranted to assess whether or not such high concentrations of FCS are required when using HES as a CPA. Moreover, it would be of interest to measure cell viability and cryopreservation efficiency by investigating other parameters, such as cell viability, recovery, and metabolism. Growth rates, cell-substrate attachment, and gene and protein analyses would also be valuable to quantify. While we did not assess these parameters in this manuscript, we are interested in building off the present study to do so with our future investigations.

## Conclusions

In sum, we show that both human keratinocytes and fibroblasts can be effectively cryopreserved using HES as a CPA. That fibroblasts can be preserved in a HES-containing solution is, to our knowledge, a novel and previously unreported finding. Given that DMSO is the most commonly used CPA and is believed to be more toxic than HES, these findings are of significance for tissue-based replacement therapies. Therapies that require the use of keratinocyte and fibroblast cells, such as those aimed at treating skin wounds or skin burns, may be optimized by substituting a portion or all of DMSO with HES during cryopreservation protocols.

## Abbreviations

CPA: Cryoprotectant agent
DMSO: Dimethyl sulfoxide
FCS: Fetal calf serum
HES: Hydroxyethyl starch

## References

[1] Griffith LG, Naughton G (2002). Tissue engineering--current challenges and expanding opportunities. Science.

[2] Metcalfe AD, Ferguson MW (2007). Tissue engineering of replacement skin: the crossroads of biomaterials, wound healing, embryonic development, stem cells and regeneration. J R Soc Interface.

[3] Brockbank KG, Heacox AE, Schenke-Layland K (2011). Guidance for removal of fetal bovine serum from cryopreserved heart valve processing. Cells Tissues Organs.

[4] Gurtovenko AA, Anwar J (2007). Modulating the structure and properties of cell membranes: the molecular mechanism of action of dimethyl sulfoxide. J Phys Chem B.

[5] Zambelli A, Poggi G, Da Prada G, Pedrazzoli P, Cuomo A, Miotti D, Perotti C, Preti P, della Cuna Robustelli G (1998). Clinical toxicity of cryopreserved circulating progenitor cells infusion. Anticancer Res.

[6] Windrum P, Morris TC, Drake MB, Niederwieser D, Ruutu T (2005). Variation in dimethyl sulfoxide use in stem cell transplantation: a survey of EBMT centres. Bone Marrow Transplant.

[7] Mueller LP, Theurich S, Christopeit M, Grothe W, Muetherig A, Weber T, Guenther S, Behre G (2007). Neurotoxicity upon infusion of dimethylsulfoxide-cryopreserved peripheral blood stem cells in patients with and without pre-existing cerebral disease. Eur J Haematol.

[8] Ruiz-Delgado GJ, Mancias-Guerra C, Tamez-Gomez EL, Rodriguez-Romo LN, Lopez-Otero A, Hernandez-Arizpe A, Gomez-Almaguer D, Ruiz-Arguelles GJ (2009). Dimethyl sulfoxide-induced toxicity in cord blood stem cell transplantation: report of three cases and review of the literature. Acta Haematol.

[9] Schlegel PG, Wolfl M, Schick J, Winkler B, Eyrich M (2009). Transient loss of consciousness in pediatric recipients of dimethylsulfoxide (DMSO)-cryopreserved peripheral blood stem cells independent of morphine co-medication. Haematologica.

[10] Rowley SD, Feng Z, Yadock D, Holmberg L, Macleod B, Heimfeld S (1999). Post-thaw removal of DMSO does not completely abrogate infusional toxicity or the need for pre-infusion histamine blockade. Cytotherapy.

[11] Hoyt R, Szer J, Grigg A (2000). Neurological events associated with the infusion of cryopreserved bone marrow and/or peripheral blood progenitor cells. Bone Marrow Transplant.

[12] Windrum P, Morris TC (2003). Severe neurotoxicity because of dimethyl sulphoxide following peripheral blood stem cell transplantation. Bone Marrow Transplant.

[13] Hequet O, Dumontet C, El Jaafari-Corbin A, Salles G, Espinouse D, Arnaud P, Thieblemont C, Bouafia F, Coiffier B (2002). Epileptic seizures after autologous peripheral blood progenitor infusion in a patient treated with high-dose chemotherapy for myeloma. Bone Marrow Transplant.

[14] Nakasato SK (1982). Evaluation of hetastarch. Clin Pharm.

[15] Wisselink W, Patetsios P, Panetta TF, Ramirez JA, Rodino W, Kirwin JD, Zikria BA (1998). Medium molecular weight pentastarch reduces reperfusion injury by decreasing capillary leak in an animal model of spinal cord ischemia. J Vasc Surg.

[16] Stolzing A, Naaldijk Y, Fedorova V, Sethe S (2012). Hydroxyethylstarch in cryopreservation - mechanisms, benefits and problems. Transfus Apher Sci.

[17] Waters LM, Christensen MA, Sato RM (1989). Hetastarch: an alternative colloid in burn shock management. J Burn Care Rehabil.

[18] Kiesewetter H, Blume J, Jung F, Spitzer S, Wenzel E (1990). Haemodilution with medium molecular weight hydroxyethyl starch in patients with peripheral arterial occlusive disease stage IIb. J Intern Med.

[19] Schmand JF, Ayala A, Morrison MH, Chaudry IH (1995). Effects of hydroxyethyl starch after trauma-hemorrhagic shock: restoration of macrophage integrity and prevention of increased circulating interleukin-6 levels. Crit Care Med.

[20] Pasch J, Schiefer A, Heschel I, Rau G (1999). Cryopreservation of keratinocytes in a monolayer. Cryobiology.

[21] Pasch J, Schiefer A, Heschel I, Dimoudis N, Rau G (2000). Variation of the HES concentration for the cryopreservation of keratinocytes in suspensions and in monolayers. Cryobiology.

[22] Maruyama M, Kenmochi T, Sakamoto K, Arita S, Iwashita C, Kashiwabara H (2004). Simplified method for cryopreservation of islets using hydroxyethyl starch and dimethyl sulfoxide as cryoprotectants. Transplant Proc.

[23] Allen ED, Weatherbee L, Spencer HH, Lindenauer SM, Permoad PA (1976). Large unit red cell cryopreservation with hydroxyethyl starch. Cryobiology.

[24] Lionetti FJ, Hunt SM (1975). Cryopreservation of human red cells in liquid nitrogen with hydroxyethyl starch. Cryobiology.

[25] Stiff PJ, Murgo AJ, Zaroulis CG, DeRisi MF, Clarkson BD (1983). Unfractionated human marrow cell cryopreservation using dimethylsulfoxide and hydroxyethyl starch. Cryobiology.

[26] Clapisson G, Salinas C, Malacher P, Michallet M, Philip I, Philip T (2004). Cryopreservation with hydroxyethylstarch (HES) + dimethylsulfoxide (DMSO) gives better results than DMSO alone. Bull Cancer.

[27] Ashwood-Smith MJ, Warby C, Connor KW, Becker G (1972). Low-temperature preservation of mammalian cells in tissue culture with polyvinylpyrrolidone (PVP), dextrans, and hydroxyethyl starch (HES). Cryobiology.

[28] Lionetti FJ, Hunt SM, Gore JM, Curby WA (1975). Cryopreservation of human granulocytes. Cryobiology.

[29] Naaldijk Y, Staude M, Fedorova V, Stolzing A (2012). Effect of different freezing rates during cryopreservation of rat mesenchymal stem cells using combinations of hydroxyethyl starch and dimethylsulfoxide. BMC Biotechnol.

[30] Naaldijk Y, Friedrich-Stockigt A, Sethe S, Stolzing A. Comparison of different cooling rates for fibroblast and keratinocyte cryopreservation. J Tissue Eng Regen Med. 2016;10(10):E354–E364. 10.1002/term.1815 23963809

[31] Terzic A, Folmes CD, Martinez-Fernandez A, Behfar A (2011). Regenerative medicine: on the vanguard of health care. Mayo Clin Proc.

[32] Johnson AA, Naaldijk Y, Hohaus C, Meisel HJ, Krystel I, Stolzing A. Protective effects of alpha phenyl-tert-butyl nitrone and ascorbic acid in human adipose derived mesenchymal stem cells from differently aged donors. Aging. 2016 [Epub ahead of print]. 10.18632/aging.101035 PMC5361667 27638293

[33] Naaldijk Y, Johnson AA, Ishak S, Meisel HJ, Hohaus C, Stolzing A (2015). Migrational changes of mesenchymal stem cells in response to cytokines, growth factors, hypoxia, and aging. Exp Cell Res.

[34] Rohani L, Johnson AA, Arnold A, Stolzing A (2014). The aging signature: a hallmark of induced pluripotent stem cells?. Aging Cell.

[35] Berz D, McCormack EM, Winer ES, Colvin GA, Quesenberry PJ (2007). Cryopreservation of hematopoietic stem cells. Am J Hematol.

[36] Gao D, Critser JK (2000). Mechanisms of cryoinjury in living cells. ILAR journal/National Research Council, Institute of Laboratory Animal Resources.

[37] Sun TT, Green H (1976). Differentiation of the epidermal keratinocyte in cell culture: formation of the cornified envelope. Cell.

[38] Abercrombie M (1978). Fibroblasts. J Clin Pathol Suppl.

[39] Muller-Schweinitzer E (2009). Cryopreservation of vascular tissues. Organogenesis.

[40] Saraste A, Pulkki K (2000). Morphologic and biochemical hallmarks of apoptosis. Cardiovasc Res.

[41] Borderie VM, Lopez M, Lombet A, Carvajal-Gonzalez S, Cywiner C, Laroche L (1998). Cryopreservation and culture of human corneal keratocytes. Investigative Ophthalmology and Visual Science.

[42] Singbartl K, Langer R, Henrich A (1998). Altered membrane skeleton of hydroxyethylstarch-cryopreserved human erythrocytes. Cryobiology.

[43] Wiedermann CJ (2008). Systematic review of randomized clinical trials on the use of hydroxyethyl starch for fluid management in sepsis. BMC Emerg Med.

[44] Rowley SD, Feng Z, Chen L, Holmberg L, Heimfeld S, MacLeod B, Bensinger WI (2003). A randomized phase III clinical trial of autologous blood stem cell transplantation comparing cryopreservation using dimethylsulfoxide vs dimethylsulfoxide with hydroxyethylstarch. Bone Marrow Transplant.

